# Development and Characterization of Biointeractive Gelatin Wound Dressing Based on Extract of *Punica granatum* Linn

**DOI:** 10.3390/pharmaceutics12121204

**Published:** 2020-12-11

**Authors:** Marismar F. do Nascimento, Juliana C. Cardoso, Tarsizio S. Santos, Lívia A. Tavares, Tatiana N. Pashirova, Patricia Severino, Eliana B. Souto, Ricardo L. C. de Albuquerque-Junior

**Affiliations:** 1 Health and Environment Post-Graduating Program, University Tiradentes (UNIT), Aracaju 49032-490, Sergipe, Brazil; marismar.fernandes@gmail.com (M.F.d.N.); juaracaju@yahoo.com.br (J.C.C.); tbiotec@hotmail.com (T.S.S.); livianjos15@hotmail.com (L.A.T.); 2 School of Nursing, University of Pernambuco, Brazil BR 203, Km 2, s/n, Petrolina 56328-903, Pernambuco, Brazil; 3 Institute of Research and Technology (ITR), Av. Murilo Dantas 300, Aracaju 49032-490, Sergipe, Brazil; 4 Department of Pharmaceutical Technology, Faculty of Pharmacy (FFUC), University of Coimbra, Pólo das Ciências da Saúde, Azinhaga de Santa Comba, 3000-548 Coimbra, Portugal; tatyana_pashirova@mail.ru; 5 Arbuzov Institute of Organic and Physical Chemistry, FRC Kazan Scientific Center of RAS, Arbuzov St., 8, 420088 Kazan, Russia; 6 School of Pharmacy, Industrial Biotechnology Post-Graduating Program, University Tiradentes, Aracaju 49032-490, Sergipe, Brazil; pattypharma@gmail.com; 7 Tiradentes Institute, 150 Mt Vernon St, Dorchester, MA 02125, USA; 8 Center for Biomedical Engineering, Department of Medicine, Brigham and Women & Hospital, Harvard Medical School, 65 Landsdowne Street, Cambridge, MA 02139, USA; 9 CEB—Centre of Biological Engineering, University of Minho, Campus de Gualtar, 4710-057 Braga, Portugal

**Keywords:** gallic acid, ellagic acid, gelatin, biological dressings, *Punica granatum*, wound healing

## Abstract

*Punica granatum* Linn (pomegranate) extracts have been proposed for wound healing due to their antimicrobial, antioxidant, and anti-inflammatory properties. In this work, we designed biointeractive membranes that contain standard extracts of *P. granatum* for the purpose of wound healing. The used standard extract contained 32.24 mg/g of gallic acid and 41.67 mg/g of ellagic acid, and it showed high antioxidant activity (the concentration of the extract that produces 50% scavenging (IC_50_) 1.715 µg/mL). Compared to the gelatin-based membranes (GEL), membranes containing *P. granatum* extracts (GELPG) presented a higher maximal tension (*p* = 0.021) and swelling index (*p* = 0.033) and lower water vapor permeability (*p* = 0.003). However, no difference was observed in the elongation and elastic modulus of the two types of membranes (*p* > 0.05). Our wound-healing assay showed that a GELPG-treated group experienced a significant increase compared to that of the control group in their wound contraction rates on days 3 (*p* < 0.01), 7 (*p* < 0.001), and on day 14 (*p* < 0.001). The GELPG membranes promoted major histological changes in the dynamics of wound healing, such as improvements in the formation of granular tissue, better collagen deposition and arrangement, and earlier development of cutaneous appendages. Our results suggest that a biointeractive gelatin-based membrane containing *P. granatum* extracts has a promising potential application for dressings that are used to treat wounds.

## 1. Introduction

Dermal injuries affect approximately 2% of the general population and compromise a quality of life; they are also responsible for up to 4% of the total expenditure on healthcare worldwide [1,2]. The healing of wounds is a dynamic process that occurs spontaneously, and it involves inflammation, granulation tissue formation, and collagen deposition and remodeling [3,4]. Despite this self-resolution, the natural healing of wounds is usually a long-term process and may result in a poor-quality contracted scar that compromises tissue function [5]. Wound care and management may therefore be necessary in ensuring that a healing process successfully occurs [6]. 

Modern wound management makes use of biointeractive membranes instead of conventional semisolid formulations, and these membranes can both adhere to the wound and work as an in-situ drug delivery system [3,4,7]. Creams, ointments, and gels do not possess such advantages because of the inconvenience posed by leakages and because of the short residence time of the drug in the wound. Furthermore, these membranes are able to provide a physical barrier, which avoids additional forming mechanical lesions and the set-up of infections [8]. 

Biocompatible and biodegradable polymers have previously been used as the matrices in formulations that are used for wound dressings. Nevertheless, for a dressing to be suitable for wound application, it needs to be flexible, durable, permeable to water vapor, and to have adequate mechanical properties to adhere to the tissue it has been applied. It must also be able to maintain a moist environment and protect the wound from infection. Wound dressings containing therapeutic agents topically released into the site of an injury can be thought of as a strategic approach in the control of inflammatory responses, the prevention of infections, and the promotion of tissue regeneration [9].

Gelatin-based membranes have been widely used as drug carrier dressings, because they release their drugs into the wounds slowly and prevent fluid from being lost due to exudation [3]. Gelatin has film-forming properties and is a biodegradable polymer, as it also allows physiological absorption to occur, thereby avoiding the trauma typically caused by removing a dressing [10].

Membranes containing bioactive molecules, such as antioxidants and anti-inflammatory drugs, have been successfully used in decreasing the amount of time it takes to heal a wound [11]. The incorporation of natural products in these membranes has become an excellent alternative both to reduce local inflammation and to aid healing [6,9,12].

Aqueous extracts of pomegranate peel have previously shown potent effects on cultures of human skin cells; they stimulate both dermal fibroblast proliferation and collagen synthesis while inhibiting major collagen-degrading enzymes [13]. Furthermore, topical applications of this extract also exhibited beneficial healing effects in rat excision wound models [14,15]. The fruit from *Punica granatum* Linn (pomegranate) is a rich source of bioactive compounds, such as tannins and a number of phenolics [16]. The peel extract of the pomegranate has a wide variety of pharmacological functions, including antibacterial, anti-inflammatory [17], and, in particular, antioxidant properties [16]. It has been reported that these extracts are able to reduce the oxidative stress in dermal tissues [18,19] and to protect the skin from damage caused by UVB irradiation-induced photoaging [20]. 

Consumer demand for pomegranates has increased significantly in Western countries due to their healthy and nutritional characteristics. As a result, products containing these components are being widely developed by the food and pharmaceutical industries. In this work, we describe the development and characterization of a membrane containing a standard aqueous extract of the fruit peel of *P. granatum* (AEPG) and evaluate its effectiveness as a dressing material in the wound-healing process of a rodent model.

## 2. Materials and Methods

### 2.1. Plant Material 

Fruits of *P. granatum* were obtained in Pernambuco, Brazil (06°45′57″ S and 42°17′17″ W). The plant was identified, and a voucher (number 20881) was deposited in the Herbarium of the Federal University of Sergipe.

### 2.2. Extraction Procedure and Sample Preparation

*P. granatum* peels were dried at 55 °C for 5 days. Five grams of dried and powdered (32-mesh) peels were extracted by the dynamic maceration process using a magnetic stirrer and water 1:100 (*w/v*) at 100 °C for 2 h. The extracts were filtered and concentrated in an airflow oven at 55 °C for 3 days. The yield of the extract obtained was calculated and expressed as a percentage.

For high-performance liquid chromatography (HPLC) analysis, the crude extract of *P. granatum* (AEPG) was dissolved in methanol:water (50:50, *v/v*) (1 mg/mL), filtered through a 0.45-µm membrane (Millipore, Merck, Billerica, MA, USA), and an aliquot of 10 μL was injected into the chromatographic system.

### 2.3. HPLC Instrumentation and Chromatographic Condition

The HPLC analysis were carried out using a Shimadzu system equipped with a LC-6AD HPLC pump, an SPD-M20A diode array detector (DAD) operated with LC Solution data station software LabSolutions DB (Shimadzu, Tokyo, Japan). Mobile phases were filtered with a nylon solvent filter (0.45 μm, Merck KGaA, Darmstadt, Germany). The water used in experiments was obtained with the Millipore (São Paulo, Brazil) Milli-Q purification system. The chromatographic separation of samples was achieved on a reversed-phase HPLC column (C18, 250 × 4.6 mm, 5 µm, Luna, Phenomenex, Torrance, CA, USA). A binary gradient elution at a flow rate of 1 mL/min was employed using an aqueous solution of phosphoric acid 0.1% (*v/v*) as solvent A and acetonitrile as solvent B, as follows: 1–5% B at 0–5 min, 5–8% B at 5–10 min, 8% B at 10–16 min, 8–25% B at 16–22 min, 25–90% B at 22–27 min, and 90–1% B at 27–33 min. The diode array detector was set at 260 nm for acquiring chromatograms. Quantification was achieved using the linear calibration curves of gallic acid (1–10 µg/mL) and ellagic acid (1–5 µg/mL) standards.

### 2.4. Antioxidant Activity

The radical scavenging activity of the extract was assessed by the free radical 2,2-diphenyl-1-picryl-hydrazyl-hydrate (DPPH) method. Samples of the hydroethanolic solutions, with concentrations between 0.1 and 15 mg/mL, were prepared. For each concentration, 3-mL aliquots were mixed with 750 µL of 400-mM DPPH solution. The mixture was then homogenized and left to stand for 15 min at room temperature without incident light. Measurements were then performed on the samples using a spectrophotometer at a wavelength of 517 nm. The experiment was performed in triplicate, and the percentage inhibition of the free radicals was calculated by %I = (AC − AA)/AC × 100. In this equation, %I is the percentage inhibition of the free radical DDPH of the sample solution as compared to the control (which was the DPPH solution), AC is the absorbance of the control solution, and AA is the absorbance of the sample solution. The IC_50_ value (i.e., the concentration of the extract that produces 50% scavenging) was calculated from the graph. The %I plotted against the concentration is expressed in µg/mL.

### 2.5. Preparation of Gelatin-Based Membrane Loaded with Aqueous Extract of P. granatum

The membranes were prepared by dispersing gelatin powder in acetic acid of 0.5 mol/L at 25 °C for 24 h. The final polymer concentration was 1%. The aqueous extract of *P. granatum* (AEPG), which was earlier solubilized in propylene glycol (plasticizer), was added to the gelatin dispersion. The concentrations of the plasticizer and the AEPG were 20% (dry basis) and 5%, respectively, of the dry polymer mass. The membranes were produced through a casting process, where the dispersion was poured into a polypropylene plate to allow the solvent to evaporate at room temperature. The membranes were produced both with and without the AEPG.

### 2.6. Characterization

#### 2.6.1. Mechanical Properties

Analyses of the mechanical properties of the membranes were conducted using a tensile strength apparatus (TA-TX2, Stable Micro Systems, Godalming, UK). Samples of the membranes were cut into strips of 20 by 10 mm. Each strip was measured with a micrometer (Mitutoyo digimatic micrometer, Kanagawa, Japan) at three points in order to determine the strips’ thicknesses. Each analysis was carried out at room temperature, and ten replicates were performed. The measured speed used was 1.0 mm/s, and the initial gauge length was 10 mm. The calculations were made through σ = F/A, where σ is the tensile strain, measured in N/mm^2^, F is the force in N, and A is the cross-sectional area in mm^2^.

#### 2.6.2. Water Vapor Transmission Rates (WVTRs)

The permeability of the membranes was determined by the water vapor loss in a gravimetric cup film sealed method under known room humidity (RH) given by saturated solutions in contact with non-dissolved salt (KBr 84% RH) placed in a desiccator containing silica in a dehumidified room. Each experiment was performed with five replicates during 48 h. The water vapor transmission was calculated by WVT = wh/A, where w is the lost mass in g, A is the membrane area in m^2^, and h is the membrane thickness in mm. The permeability was calculated by P = WVT/Δpt, where t is time in days, and Δp is the difference between the internal and external relative humidity pressures.

#### 2.6.3. Swelling Index

Studies into the swelling of the membrane were conducted using a neutral environment (phosphate-buffered saline (PBS), pH 7.2). Each membrane sample (surface area 2 × 3 cm^2^) was preweighed before and submerged into a 30-mL medium in a plastic container. The weight of the film was determined at predetermined intervals after the removal of excess surface water. The test was done in triplicate. The degree of swelling was calculated by I% = (Wt × 100)/Wo, where Wt is the weight of the membrane at time t, and Wo is the weight of the dry membrane. 

#### 2.6.4. Infrared Spectroscopy (FTIR)

Infrared absorption data of the membranes was obtained in the range 4000–400 cm^−1^ using an FTIR spectrophotometer (model Spectrum BX, Perkin Elmer, Bucks, UK) at room temperature.

#### 2.6.5. Thermal Analysis

Differential scanning calorimetry (DSC) curves were obtained by a DSC-50 cell (Shimadzu, Kyoto, Japan) using aluminum crucibles. Approximately 2 mg of samples were put into these crucibles, and the measurements were made under a dynamic nitrogen atmosphere (50 mL/min) at a heating rate of 10 °C/min in the temperature range 25–600 °C. The DSC cell was calibrated with indium (melting point (m.p.) = 156.6 °C and fusion enthalpy (ΔHfus.) = 28.54 J/g) and zinc (m.p. = 419.6 °C). Thermogravimetric (TG/DTG) curves were obtained with the TGA-50 thermobalance (Shimadzu, Kyoto, Japan) using platinum crucibles that contained roughly 3 mg of a sample. These measurements were made under a dynamic nitrogen atmosphere (50 mL/min) at a heating rate of 10 °C/min in the temperature range 25–900 °C.

#### 2.6.6. Color Measurement

The color values of the membranes were measured with a chromameter (CR-300 chromameter, Minolta Camera Co., Osaka, Japan). The membrane specimens were placed onto the surface of a white standard plate (calibration plate CR-A43, Minolta Camera Co., Osaka, Japan), and the *L*, *a*, and *b* color values were measured. The total color difference (Δ*E*) was calculated by

$$\Delta E=\;\sqrt{{\left(L-L^{’}\right)}^{2}+{\left(a-a^{’}\right)}^{2}+{\left(b-b^{’}\right)}^{2}}$$

where the L′, a′, and b′ values are the Hunter color values of the standard white plate (L′ = 74.1, a′ = 6.1, and b′ = 13.1).

#### 2.6.7. Scanning Electron Microscopy (SEM)

The membranes were mounted onto aluminum stubs and coated with a thin layer of gold. They were then visualized with a JEOL Model JSM-6360-LV (Tokyo, Japan) scanning electron microscope at an accelerated voltage of 20 kV.

### 2.7. Wound-Healing Assay

#### 2.7.1. Animals 

The animals used in this study were adult male *Rattus norvegicus albinus* of the Wistar lineage and weighed 250–300 g. The specimens were handled according to the ethical principles in animal research that were adopted by the Brazilian College of Animal Experimentation, and they were supplied with food and water ad libitum. Experimental protocols and procedures were approved by the University Tiradentes Animal Care and Use Committee (CEUA no. 030811, 21 October 2015).

#### 2.7.2. Surgical Procedures and Groups 

Sixty specimens were anesthetized with intraperitoneal ketamine-xylazine (100–5 mg/kg), and standard-sized round-shaped surgical wounds were made in their backs using a metallic punch of 8.0 mm. The specimens were handled in accordance with the principles of the aseptic chain in order to avoid bacterial contamination. The specimens were randomly assigned into three groups of 20:CTR, which was a control group with undressed wounds, GEL, a group whose wounds were dressed with gelatin-based membranes, and GELPG, whose wounds were dressed with gelatin-based membranes containing AEPG. After 3, 7, 14, and 21 days, five animals in each group were euthanized in a CO_2_ chamber, and the healing/scar area was surgically removed, formalin-fixed, and paraffin-embedded.

#### 2.7.3. Assessment of the Wound Closure Rates (WCR)

After 3, 7, 14, and 21 days, craniocaudal and laterolateral measures of each wound were assessed using a digital caliper (precision 0.01 mm) prior to the excision of the wounds. The final wound areas (A) were obtained by A = πRr, where R represents the craniocaudal axis and r the laterolateral axis of the wounds. The clinical features of the wounds were also monitored, so as to observe the presence of a crust, secretion, necrosis, or hypertrophic scarring.

#### 2.7.4. Histological Procedures and Morphological Analysis 

A series of 5-µm-thick sections were obtained from the paraffin-embedded samples and stained using hematoxylin-eosin (HE). The intensity of the acute inflammatory infiltrate was assessed in day 3 as follows: 0 = absence of inflammatory infiltrate, 1 = inflammatory cells representing 10% of the cell population, 2 = inflammatory cells representing between 10% and 50% of the cell population, and 3 = inflammatory cells representing more than 50% of the cell population. The inflammatory profile of the samples was classified as either being acute (predominance of polymorphonuclear cells) or chronic (predominance of mononuclear cells) [6]. The histological grading of the wound healing was assessed on days 7, 14, and 21 using a semiquantitative scoring system based on an ordinal scale considering six histological parameters related to the healing process [21,22], as demonstrated in Table 1. Three histological fields in each histological section were selected (one from each edge, and one from the center of the wounds, 100× magnification), photomicrographed, and analyzed. The total healing score in each group was calculated by adding the scores of the individual criterion in all the histological fields. The data obtained in this analysis were expressed as mean ± standard deviation (SD). In order to assess the collagen deposition and provide a descriptive analysis, histological sections were stained using Sirius Red and analyzed under polarized light. Collagen fibers were analyzed according to their birefringence pattern (greenish/yellow-greenish or orange or orange-reddish); morphological appearance (wavy or stretched, thin or thick, or short or long); and architectural arrangement (reticular, parallel, or interlaced). All of the readings were performed by investigators that were unaware of the type of treatment administered to the specimens (six histological sections/animal).

### 2.8. Statistical Analysis 

The data was analyzed using ANOVA followed by a Tukey’s test. The analysis of the inflammatory infiltrate was done according to the Kruskal–Wallis test followed by a Dunn’s test. *p*-values of less than 0.05 were considered significant.

## 3. Results and Discussion

In these studies, the extracts were topically administered either while raw or as a formulated ointment. However, this is the first report assessing the healing effects of extracts of *P. granatum* incorporated into gelatin-based membranes. 

### 3.1. Characterization of the Pomegranate Peel Extracts

**Figure 1:**
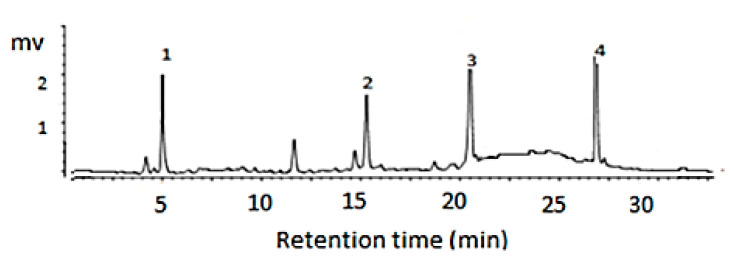
Chromatography of the aqueous extract of *Punica granatum* L. The peaks represent gallic acid [1], punicalagin α [2], punicalagin β [3], and ellagic acid [4] at 260 nm.

The major chemical compounds identified by the HPLC-DAD in the dry extracts were punicalagin α and β, ellagic acid, and gallic acid (Figure 1). The ellagic and gallic acids were quantified using an external standard method, and the ellagic acid was found to be at a higher concentration (Table 1). These compounds are classified as high-polarity tannins, which makes them easier to extract using polar solvents. Romani et al. (2012) [23] identified the same chemical constituents of aqueous *P. granatum* L. extracts in their work.

The set-up of the concentration of chemical markers for a specific biological activity is mandatory when obtaining a standard formulation [24]. As such, AEPG containing 32.24 mg/g of gallic acid and 41.67 mg/g of ellagic acid were used for preparing the dressings that would be used for wound healing (Table 1). 

We found that AEPG presented high-antioxidant activity at very low concentrations, showing an IC_50_ of 1.715 ± 0.24 µg/mL. Since Aqil et al. (2012) [25] found a higher IC_50_ of pomegranate, around 17 µg/mL, it seems that our sample presents strong antioxidant activity. Furthermore, the antioxidant activity found in the current study was greater than that which has already been reported [26], using extracts from leaves (200–330 µg/mL); this suggests that extracts from peels have greater antioxidant activity.

### 3.2. Characterization of the Membranes

According to Peh et al. (2000) [27], membranes used for wound healing are supposed to be strong and flexible. The mechanical properties of GEL and GELPG are presented in Table 2. The GELPG membranes had higher tensile strength than the GEL membranes did (*p* = 0.021), but no differences were observed regarding their elongation and Young’s modulus (*p* > 0.05). The incorporation of the extract into the membrane formulation increased the force necessary to break the membrane, but the elasticity (Young’s modulus) and the elongation at break remained similar to that of the original formulation; this indicates that the GELPG is a flexible membrane.

Color alteration is a parameter that can aid in determining the quality of a membrane, since color shows the homogeneity and stability of the final formulation and is an indicator of the presence of ellagitannin compounds [27]. The GELPG membranes presented a higher value of Δb = 10.56 than the GEL membranes did, which had Δb = −12.30 (Table 2). Since positive values of this parameter indicate yellowness, it is possible to suggest that the ellagitannins are the compounds responsible for the yellow color. However, no difference was observed in the △a and △L values, which indicates that the membranes did not change in terms of their greenish and transparency characteristics, respectively [25].

Wound dressings are supposed to maintain a moist environment in the healing area. Hydrocolloids are able to form gels upon contact with wound exudates, and these allow fluid leakage without wound dehydration [10]. The GELPG membranes exhibited a higher swelling index than that of the GEL membranes (*p* = 0.033). The water retention of the system after swelling occurred is related to interactions between the exudate fluids and polar groups from the polymer, plasticizer agent, and active substances from the extract. The pH of the exudative fluids of chronic wounds tend to be alkaline or neutral when compared to the intact surrounding skin. However, acid compounds from the GELPG membranes can neutralize or acidify the microenvironment, increasing the water absorption. At pH 7.0, the hydroxyl groups are partially ionized, avoiding the excessive water uptake and, consequently, the instantaneous dissolution of the membrane. This modulation in the water uptake favors the water retention capability. Despite showing higher swelling behavior, GELPG membrane dissolution was not observed. Bigi et al. (2004) [28] demonstrated that gelatin membranes that are immersed in a physiological solution have a swelling index of around 300% after five min. The GELPG and GEL membranes in physiological pH solutions showed swelling indexes of 412.5% and 253.2%, respectively, after three h. In addition to the water–polymer interaction favored by the third-dimension conformational of the gelatin molecule, the incorporation of hydrophilic molecules, such as ellagitannins, increased the swelling potential of the membrane [29]. 

We observed that the permeability of GELPG was significantly lower than of GEL (*p* = 0.003) (Table 2); however, the swelling index was higher. Structural changes caused by the swelling of the hydrophilic membrane may cause internal tension, which can influence the permeation of the water vapor. Interactions between the water molecules and the polar compounds present in GELPG probably trapped the water vapor inside the membrane (favoring swelling), and this caused the diffusion rate of the water molecules throughout the membrane to be low [30]. The inclusion of molecules with a high molecular weight, such as punicalagin (MW 1084.71 g/mol), in the membrane could be responsible for the steric effect, which would decrease the membrane’s permeability. 

**Figure 2:**
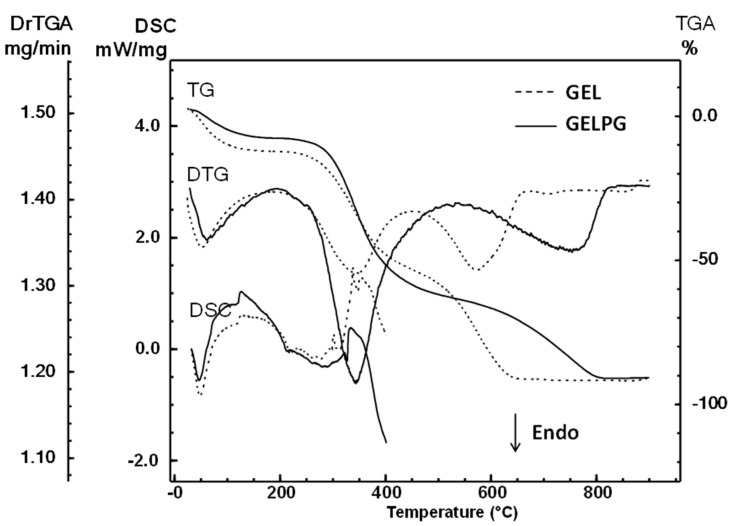
Differential scanning calorimetry (DSC), thermogravimetric (TGA), and derivative thermogravimetric (DrTGA) curves of the GEL (gelatin membrane) and GELPG (gelatin membrane containing aqueous extract of *P. granatum*).

The DSC and TG/DTG curves obtained from the analysis of the GEL and GELPG membranes are shown in Figure 2. These curves indicate that the thermal decomposition of GEL occurs at three temperature ranges, and at each, some weight is lost: 25–173 °C (Δm = 13.9%), 218–444 °C (Δm = 40.7%), and 444–675 °C (Δm = 38.3%). However, the TG/DTG curves of GELPG showed three steps of weight loss: 25–173 °C (Δm = 9.8%), 218–530 °C (Δm = 55.5%), and 530–819 °C (Δm = 27.7%). The first stage, observed up to around 173 °C, is related to the loss of adsorbed and bound water. The degradation process of GEL and GELPG occurs at roughly between 173 and 500 °C, and it is followed by carbonization. The DSC curves of the membranes exhibit similar behaviors.

The main difference between the membranes is in the percentage of mass loss. The control sample membrane exhibited a high mass loss percentage in the first stage and the lowest percentage in the second stage. The membrane loaded with pomegranate extract showed a percentage of water loss of 9.8%, while the GEL membrane lost 13.9%. Furthermore, we found that GELPG had a higher loss due to decomposition (55.5%) compared to GEL (40.7%). These results suggest that the extract was incorporated effectively.

**Figure 3:**
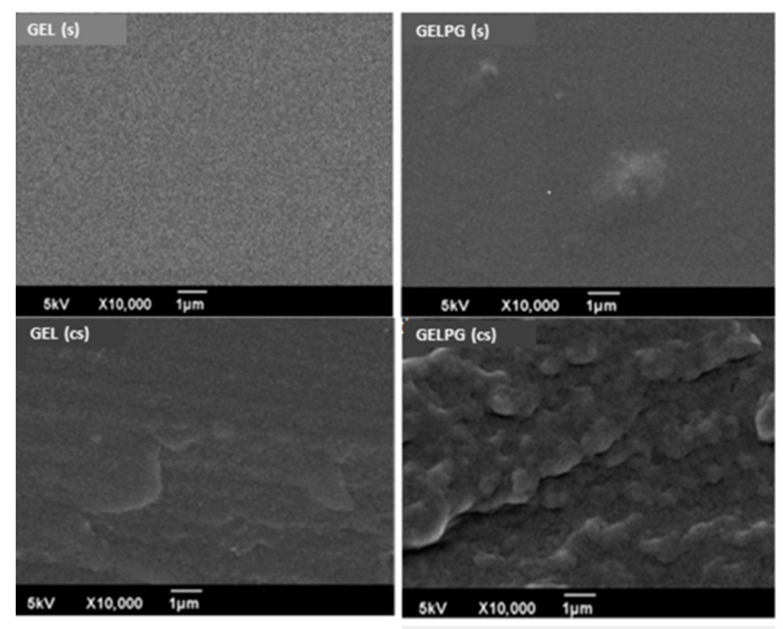
Photomicrographs of GEL and GELPG membranes. (s) are the surface images, and (cs) are the cross-section images.

Figure 3 shows photomicrographs of the surface (S) and the cross-section (CS) of the GEL and GELPG membranes. GEL had a homogeneous surface with a nano-roughness that gave it a “ground glass-like” appearance. GELPG, however, had a more irregular appearance, with some “hills” on its surface. In the CS photomicrographs, irregular “pocket-like” structures were observed in both membranes, but they were markedly more apparent in GELPG than in GEL. The interaction between water and the gelatin moved the polymer chains away from each other and thereby created “pocket-like” spaces that could contain water molecules. It is possible that the incorporation of the ellagitannin compounds that were present in the standard extract into the gelatin matrix might have enhanced the numbers and sizes of such spaces due to the highly hydrophilic nature of the final product.

**Figure 4:**
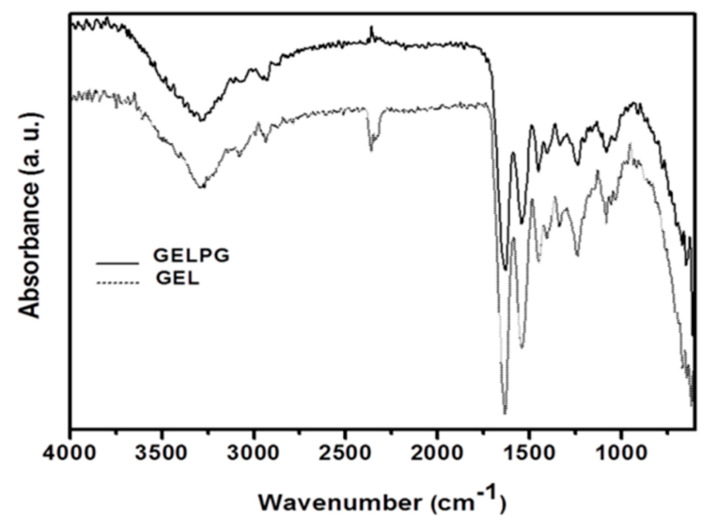
FTIR spectra of the GEL (dash line) and GELPG membranes (solid line).

The FTIR absorption bands of gelatin appear at 1653 cm^−1^, 1546 cm^−1^, and 1236 cm^−1^ and are attributed to the presence of the amide I, II, and III groups, respectively [31]. In Figure 4, the FTIR bands of GEL and GELPG were lower and occurred at 1642 cm^−1^ and 1535 cm^−1^. The absorption band around 3241 cm^−1^ (which is attributed to the stretching vibration of the N–H group bonded to the O–H group) was also shifted to a lower wave number (3411 cm^−1^); this suggests that there was an increase in the hydrogen bonding [31]. These changes suggest the occurrence of intermolecular interactions between the gelatin and the chemical compounds present in AEPG. Moreover, there was no new absorption band in the GELPG spectrum, which suggests that there was no obvious chemical reaction between the extract compounds and the gelatin.

### 3.3. Assessment of the Wound Closure Rates (WCR)

**Figure 5:**
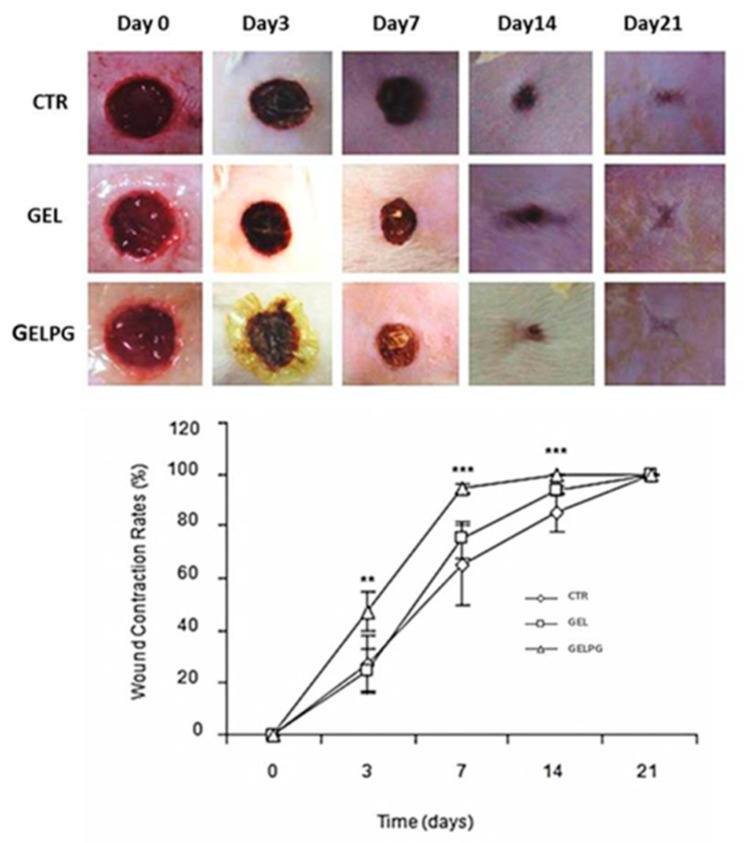
Macroscopic features of the wounds over a course of time, showing no abscess or hypertrophic/atrophic scar formation. A notable feature here is the retraction of the wound areas over time. The evolution of the wound contraction rates in the different experimental groups over time is shown here. ** GELPG is significantly different from CTR (*p* < 0.01), and *** GELPG is significantly different from CTR (*p* < 0.001).

Figure 5 presents the macroscopic features of the wounds over time, and no sign can be seen of either abscess formation in the early phases (three and seven days) or hypertrophic scars in the final ones (14 and 21 days). The wound closure rate (WCR) observed with GELPG was significantly higher than for the CTR and GEL at 3 (*p* < 0.01 and 0.001), 7 (*p* < 0.001 and 0.01), and 14 days (*p* < 0.001 and 0.001). Both the macroscopic appearance of wounds and the WCR are regarded as important parameters for evaluating the success of wound repair [6,12]. Our results suggest that the GELPG dressing is biocompatible, nontoxic, and that it improves wound healing. Other plant extracts rich in antioxidant compounds, such as gallic and ellagic acids, have also demonstrated to improve wound closure [32,33], but the precise mechanism underpinning the effect of the AEPG on the WCR is not yet clear. The wound closure process is mediated by two major pathophysiological events: wound contraction and epithelization [34]. Wound contraction has been associated with the activity of myofibroblasts; increased WCR, therefore, suggests that AEPG possibly plays a stimulatory role in myofibroblast differentiation. Although in vitro studies have demonstrated that ellagic acid inhibits both the alpha smooth muscle actin (α-SMA) expression and trans-differentiation of fibroblasts into myofibroblast-like cells [35], there is very little information about the role played by the other major compounds in AEPG (gallic acid and punicalagin α and β) on myofibroblast differentiation. In addition, re-epithelization is also essential to re-establish the epidermal epithelial barrier and provide wound closure. In fact, GA, one of the major compounds found in AEPG, has proved not only to accelerate the cell migration of keratinocytes in both normal and hyperglucidic conditions but, also, to activate hallmarks factors of wound healing, such as focal adhesion kinases (FAK), c-Jun N-terminal kinases (JNK), and extracellular signal-regulated kinases (Erk) [36]. On the other hand, punicalagin, also found in high contents in AEPG, has been shown to prevent keratinocyte death induced by reactive species of oxygen (ROS) [37], which could be helpful to sustain the cell viability of keratinocytes in the edges of wounds even under oxidative stress conditions, such as during the inflammatory phase of wound healing. The role played by the paniculum carnosum (PC) in wound closure should also be discussed. PC consists of a thin layer of striated muscle that is intimately attached to the skin and fascia of most mammals. However, further investigation is needed to clarify what the biological effects of the major chemical compounds of AEPG are that improve WCR in this manner. Furthermore, in order to reduce the influence of the panniculus carnosus (PC) on wound closure in mice, it was surgically removed in the current experimental model. PC is an extensive layer of striated muscle just beneath the subcutaneous fat present in murine skin that allows the skin to move independently from deeper muscles, and it has been associated with rapid skin contractions upon being wounded. As PC is largely absent in humans, it was removed in the current study in order to reduce its influence on wound contractions [38]. Thus, wound healing was fully dependent on epithelialization, cell proliferation, and angiogenesis, which closely mirror the biological processes of human wound healing.

### 3.4. Morphological Analysis of the Wound-Healing Process

**Figure 6:**
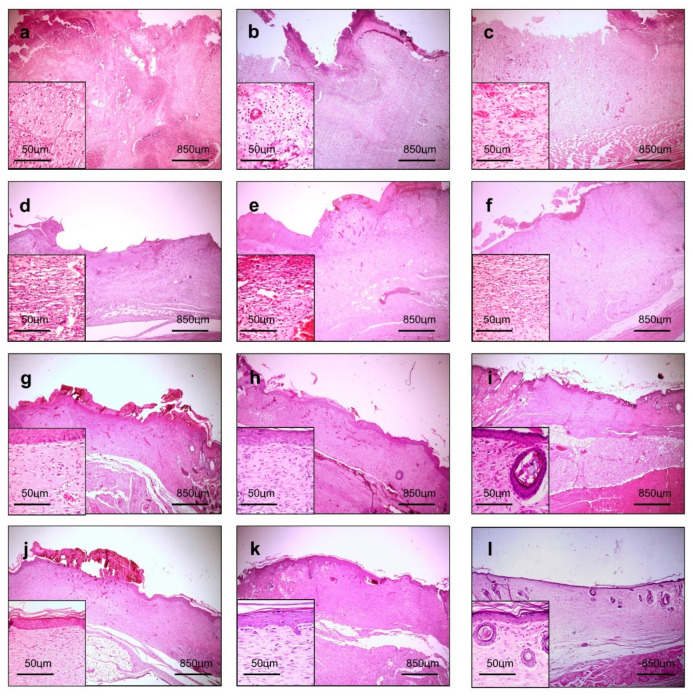
Histological sections of the groups (wounded areas) of the wound-healing biological assay over a course of time. An intense inflammatory response and edema are observed in the CTR (**a**) and GEL (**b**), while immature granulation tissue is observed in GELPG at day 3 (**c**). Well-vascularized granulation tissue is observed in the CTR (**d**) and GEL (**e**) at day 7, while fibrous connective tissue was formed in GELPG (**f**). Primary fibrous scars can be seen in all of the groups at day 14 (**g**–**i**), though hair follicles already began forming in GELPG (I). Dense fibrous connective tissue is seen in all the groups at day 21 (**j**–**l**), with multiple hair follicles observed in GELPG (**l**) (HE, 40/400× magnification).

In all the groups, there was an acute inflammatory infiltrate, particularly rich in polymorphonuclear neutrophils (PMN) and marked interstitial edema at day three (Figure 6a–c), which is consistent with the acute inflammatory phase of wound healing. In fact, a substantial amount of PMN migrate into the wounded area after injury to prevent the invasion and proliferation of microorganisms. Subsequently, PMN are progressively replaced by mononuclear cells as the wound healing continues in a bottom-top process [39]. The continued recruitment of active neutrophils occurs not only in response to the activation of the complement system and mast cells degranulation, but it is also determined by bacterial degradation products [40]. Prolonged acute inflammation impairs wound healing, because PMN release a large amount of highly active antimicrobial substances, such as reactive oxygen species (ROS), cationic peptides, and proteases, at the wound site, so that the longer the acute inflammatory phase, the more intense the PMN-induced tissue damage and the more delayed wound healing [41]. As demonstrated in Table 3, the CTR presented significantly more PMN infiltrate than the GEL and GELPG (*p* < 0.05 and *p* < 0.01, respectively). Therefore, these data suggest that the presence of gelatin-based membranes covering the wound surface might work as a physical barrier that minimizes wound contamination and reduces PMN infiltration. The decrease in the polymorphonuclear neutrophils is commonly followed by a simultaneous reduction of M1 macrophages (able to secrete proinflammatory cytokines), whereas the M2 macrophages and lymphocytes remain in the wounded area at the very beginning of the proliferative phase. This phenotype transition of the inflammatory infiltrate plays an important role in wound healing because of the extensive array of growth factors that have the potential to modulate the healing process, including fibroblast proliferation and granulation tissue formation [42]. The most important growth factors for wound healing are: (i) epithelial growth factor (EGF) and platelet-derived growth factor (PDGF), cytokines responsible for re-epithelization, (ii) fibroblast growth factor (FGF) and transforming growth factor (TGF-b), which stimulate fibroblast proliferation and myofibroblast differentiation, in addition to collagenization, and (iii) vascular endothelial growth factor (VEGF), which acts on angiogenesis and tissue granulation, particularly in the early stages of wound healing [43]. On the other hand, granulation tissue formation and progressive collagenization were seen from days 7 to 21 (Figure 6d–f), which allowed the assessment of the histological grading of wound healing, based on the criteria previously reported by Gupta and Kumar (2015) [21] and by Sultana et al. (2009) [22]. GELPG improved histological wound healing on days 7 (*p* < 0.05), 14 (*p* < 0.01), and 21 (*p* < 0.010) in comparison to the CTR. Neovascularization in the bottom of the wounds was observed earlier for the GELPG samples, occurring on day three, and more collagenized granulation tissue was seen on day seven for the GELPG samples. In addition, GELPG also exhibited an earlier development of epithelial buddings consistent with rudimentary skin appendages (on day 14) compared to the CTR and GEL (day 21). These outcomes suggest that the GELPG membranes promoted major histological changes in the dynamics of wound healing. This data may have occurred, at least in part, due to the anti-inflammatory effects of the peel-derived AEPG [44]. Furthermore, in vitro and in vivo studies have demonstrated the potent anti-inflammatory activity induced by the major chemical compounds of the extract, including ellagic acid [45], gallic acid [46], and punicalagin [47]. Since the extract presented high antioxidant properties, it is also possible to suppose that there is a relationship between the improvement in histological wound healing and a reduction of the oxidative stress in the injured tissues. In fact, oxygen-free radicals derived from activated neutrophils are supposed to both induce oxidative stress-related tissue damage and delay the healing of a wound [48]. Nevertheless, it has previously been reported that gallic acid augments the local levels of prostaglandins (PGs) during acute neutrophil-rich inflammatory responses, and such an enhancement is known to inhibit the generation of neutrophils-mediated free radicals [49]. Furthermore, ellagic acid has been reported to reduce the oxidative stress induced by other types of dermal injuries, such as UVA radiation [50] and isoproterenol [51]. Therefore, it is possible that oxidative stress was assuaged in the wounds that were dressed in the GELPG membranes. 

### 3.5. Morphological Analysis of the Collagenization

**Figure 7:**
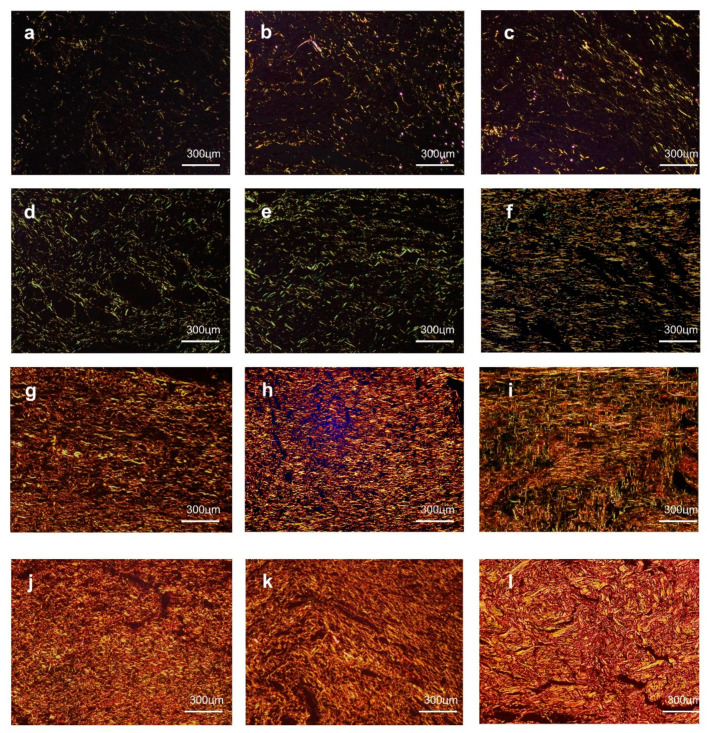
At day 3, a small amount of greenish type III collagen deposits were observed in the CTR (**a**), GEL (**b**), and GELPG (**c**). At day 7, reticular type III collagen was seen in the CTR (**d**) and GEL (**e**), while parallel arrangements of type I collagen were seen in GELPG (**f**). At day 14, dense parallel arrangements of type I collagen were observed in the CTR (**g**) and GEL (**h**), whereas, in GELPG (**i**), the fibers were slightly interlaced. At day 21, gross, interlaced type I collagen fibers were seen in all of the groups, but they were less compact in the CTR (**j**) and GEL (**k**) than in GELPG (**l**) (Sirius Red/polarized light, 400× magnification).

On day three, all of the groups presented a very small amount of deposits of thin and delicate reticularly arranged collagen fibrils with a greenish birefringence, which was consistent with those of a type III collagen (Figure 7a–c). After seven days, although all of the groups showed a marked increase in collagen contents, the fibrils remained reticularly arranged in the CTR (Figure 7d) and GEL (Figure 7e) groups, but they had a parallel arrangement in the GELPG (Figure 7f) samples. Moreover, the fibrils with greenish birefringence were predominant in the CTR (Figure 7d) and GEL (Figure 7e) samples, whereas some yellow and orange fibrils, compatible with those of type I collagen, were seen in GELPG (Figure 7e). After 14 days, type III collagen was fully replaced by type I collagen in all studied groups. The fibers had a parallel arrangement in the CTR (Figure 7g) and GEL (Figure 6h) but were interlaced in GELPG (Figure 7i). At day 21, all the groups exhibited a dense, interlaced network of gross type I collagen. Nevertheless, the fibers appeared to be thicker and grosser in GELPG and, therefore, resembled the normal appearance of dermal collagen (Figure 7l). 

Based on these histological parameters, the GELPG membranes induced a more regular and denser collagen arrangement, which suggests that the collagen deposition and remodeling was faster. Our findings are supported by works that found that topical treatments with an aqueous extract of *P. granatum* L. promoted a significant increase in the collagenization rates and stabilization of the collagen fibers in the dermal wounds of rats [1,52]. Nevertheless, although we provided evidence that AEPG may affect fibroblastic dynamics, it is important to highlight that no gross or morphological signs of collagen overproduction were observed in either the macroscopic or microscopic analysis; such overproduction can result in hypertrophic scar formation. 

The earlier development of cutaneous appendages that were observed in the GELPG group suggests that the chemical compounds of the extract may play a role in the proliferation and differentiation of epidermal cells into cutaneous appendages. Our findings are supported by studies that found that tannic acids induce keratinocyte proliferation in vitro [53]. In addition, it was already established that the increased activity of heme-oxygenase 1 (HO-1), an enzyme whose upregulated activity is closely related to that of the antioxidant effects of different chemical compounds, is able to induce keratinocyte proliferation [54]. Thus, we hypothesize that the improvement in the epithelization and earlier reconstitution of the cutaneous appendages may also be related to the antioxidant effects of AEPG, which were incorporated into the dressing membranes.

## 4. Conclusions

We demonstrated that a standard extract of *P. granatum* Linn, containing 32.24 mg/g of gallic acid and 41.67 mg/g of ellagic acid, exhibits a potent antioxidant effect, and the incorporation of this natural product into gelatin-based dressing membranes successfully improved the healing of wounds. We therefore suggest that dressing membranes containing this extract are a promising product for the treatment of chronic wounds.

## Figures and Tables

**Table 1:** Concentrations (mg/g) of the compounds present in the aqueous extract of *Punica granatum* L.

Compound	Regression Equation	R^2^	mg/g Extract
Gallic acid	*y* = 1.234*x* + 24.578	0.9998	32.24
Ellagic acid	*y* = 2.109*x* + 13.875	0.9998	41.67

**Table 2:** Characterization of the membranes.

Analysis	Parameters	GEL	GELPG	*p*-Values *
Mechanical properties	Young modulus (MPa)	633.3 ± 128.5	639.3 ± 82.0	0.917
	Elongation (%)	7.1 ± 2.5	8.7 ± 2.4	0.178
	Maximal tension (MPa)	43.3 ± 9.1	54.6 ± 7.3	0.021
Colorimetry	ΔE	18.6 ± 0.6	15.0 ± 2.8	0.162
	Δa	−3.9 ± 0.2	−3.7 ± 0.4	0.557
	Δb	−12.3 ± 0.9	10.6 ± 4.1	0.011
	ΔL	13.3 ± 1.0	9.7 ± 0.6	0.006
Swelling behavior	Swelling index (%) after 3 h/pH 7.2	253.2 ± 23.9	412.5 ± 54.2	0.033
Barrier properties	Permeability (g∙mm/d∙m^2^∙KPa)	14.0 ± 1.9	12.4 ± 1.3	0.003

* *p*-values < 0.05 represent a significant difference. Student’s *t*-test. GEL: gelatin-based membranes and GELPG: gelatin-based membranes containing *P. granatum* extracts.

**Table 3:** Histological assessment of the inflammatory response intensity scores (mean ± standard deviation) over the course of time of the healing of the wound.

Groups	Experimental Time (days)			
	Inflammation ^a^	Wound-Healing Histological Grading ^b^		
	Day 3	Day 7	Day 14	21
**CTR**	3.0 ± 0.0	9.4 ± 1.2	12.8 ± 1.7	16.3 ± 1.4
**GEL**	2.6 ± 0.5 *	10.2 ± 1.6	13.6 ± 1.9	17.1 ± 1.3
**GELPG**	2.4 ± 0.5 *	11.7 ± 1.3 *	15.1 ± 1.5 **	18.2 ± 1.2 **

^a^ Nunes et al. (2011) [6]. ^b^ Sultana et al. (2009) and Gupta and Kumar (2015) [21]. Significant differences compared with the CTR are expressed as * *p* < 0.05, ** *p* < 0.01, during the same times (columns).
